# Antimicrobial Activity of Lignin and Lignin-Derived Cellulose and Chitosan Composites against Selected Pathogenic and Spoilage Microorganisms

**DOI:** 10.3390/polym11040670

**Published:** 2019-04-11

**Authors:** Abla Alzagameem, Stephanie Elisabeth Klein, Michel Bergs, Xuan Tung Do, Imke Korte, Sophia Dohlen, Carina Hüwe, Judith Kreyenschmidt, Birgit Kamm, Michael Larkins, Margit Schulze

**Affiliations:** 1 Department of Natural Sciences, Bonn-Rhein-Sieg University of Applied Sciences, von-Liebig-Str. 20, D-53359 Rheinbach, Germany; abla.alzagameem@h-brs.de (A.A.); stephanie.klein@h-brs.de (S.E.K.); michel.bergs@h-brs.de (M.B.); xuan-tung.do@h-brs.de (X.T.D.); mclarki2@ncsu.edu (M.L.); 2 Faculty of Environment and Natural Sciences, Brandenburg University of Technology BTU Cottbus-Senftenberg, Platz der Deutschen Einheit 1, D-03046 Cottbus, Germany; b.kamm@kplus-wood.at; 3 Rheinische Friedrich Wilhelms-University Bonn, Katzenburgweg 7-9, D-53115 Bonn, Germany; i.korte@uni-bonn.de (I.K.); sophia.dohlen@uni-bonn.de (S.D.); chuewe@uni-bonn.de (C.H.); j.kreyenschmidt@uni-bonn.de (J.K.); 4 Kompetenzzentrum Holz GmbH, Altenberger Strasse 69, A- 4040 Linz, Austria; 5 Department of Forest Biomaterials, North Carolina State University, 2820 Faucette Drive Biltmore Hall, Raleigh, NC 27695, USA

**Keywords:** antimicrobial activity, antiradical activity, chitosan, hydroxypropylmethylcellulose, lignin, pathogenic microorganisms, organosolv

## Abstract

The antiradical and antimicrobial activity of lignin and lignin-based films are both of great interest for applications such as food packaging additives. The polyphenolic structure of lignin in addition to the presence of O-containing functional groups is potentially responsible for these activities. This study used DPPH assays to discuss the antiradical activity of HPMC/lignin and HPMC/lignin/chitosan films. The scavenging activity (SA) of both binary (HPMC/lignin) and ternary (HPMC/lignin/chitosan) systems was affected by the percentage of the added lignin: the 5% addition showed the highest activity and the 30% addition had the lowest. Both scavenging activity and antimicrobial activity are dependent on the biomass source showing the following trend: organosolv of softwood > kraft of softwood > organosolv of grass. Testing the antimicrobial activities of lignins and lignin-containing films showed high antimicrobial activities against Gram-positive and Gram-negative bacteria at 35 °C and at low temperatures (0–7 °C). Purification of kraft lignin has a negative effect on the antimicrobial activity while storage has positive effect. The lignin release in the produced films affected the activity positively and the chitosan addition enhances the activity even more for both Gram-positive and Gram-negative bacteria. Testing the films against spoilage bacteria that grow at low temperatures revealed the activity of the 30% addition on HPMC/L1 film against both *B. thermosphacta* and *P. fluorescens* while L5 was active only against *B. thermosphacta*. In HPMC/lignin/chitosan films, the 5% addition exhibited activity against both *B. thermosphacta* and *P. fluorescens.*

## 1. Introduction

Due to the environmental problems caused by nonbiodegradable synthetic plastic packaging materials, research has been focused to develop biodegradable packaging materials using renewable resources and biomass-derived waste. Thus, high strength oxygen-barrier films prepared from renewable forestry product waste (hot-water wood extract) were reported by Cheng et al. as an industrial, scalable, simple, and green processing approach [1]. Natural polysaccharides such as starch, cellulose, and hemicellulose have been intensively investigated as appropriate raw materials for the development of novel biodegradable food packaging materials [2,3,4,5]. In this context, the search for appropriate starting compounds is supported by the establishment of biorefineries to exploit lignocellulose feedstock (LCF) and corresponding LCF-rich biomass and/or waste [6,7,8].

Besides cellulose and hemicellulose, lignin attracts scientific interest as source of aromatic compounds, representing 30% of all non-fossil organic carbon on earth. Lignin is produced in large quantities as a byproduct of the pulp and paper industry wherein it is primarily burnt as a low-efficiency fuel to power paper mills [9]. Aside from its abundance and inexpensive supply, lignin is favorable for its numerous attractive properties, such as biodegradability, antioxidant activity, high carbon content, high thermal stability, and stiffness, comprehensively reviewed by Rinaldi et al. [10]. These important features of lignin can be synergistically combined with the advanced functionalities of well-defined polymers via covalent bond linkages [11]. Concerning the antibacterial properties of lignin, it seems to be also a promising green replacement for fossil-based agents useful against several dangerous microorganisms. Its biocidal activity makes it a more attractive compound than silver nanoparticles due to its reduced environmental impact. Some studies have shown that lignin has an antimicrobial effect [12]. The phenolic hydroxyl and methoxy groups contained in lignin have been reported to be biologically active. Depending on biomass source and pulping process, lignins vary in their 3D structure and possess different antimicrobial, antioxidant, and UV absorption properties. Various investigations have suggested that lignins can be applied to stabilize food and feedstuffs because of their antioxidant, antifungal, and antiparasitic properties [12,13,14,15,16]. Thus, Guo and others found that lignin extracts show considerable antimicrobial activity against *Listeria innocua* (a Gram-positive bacteria) [17].

**Figure 1:**
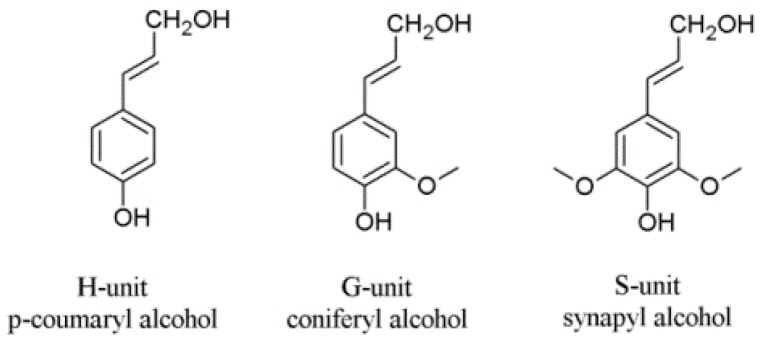
Lignin units H, G, and S derived from corresponding cinnamoyl alcohols.

**Figure 2:**
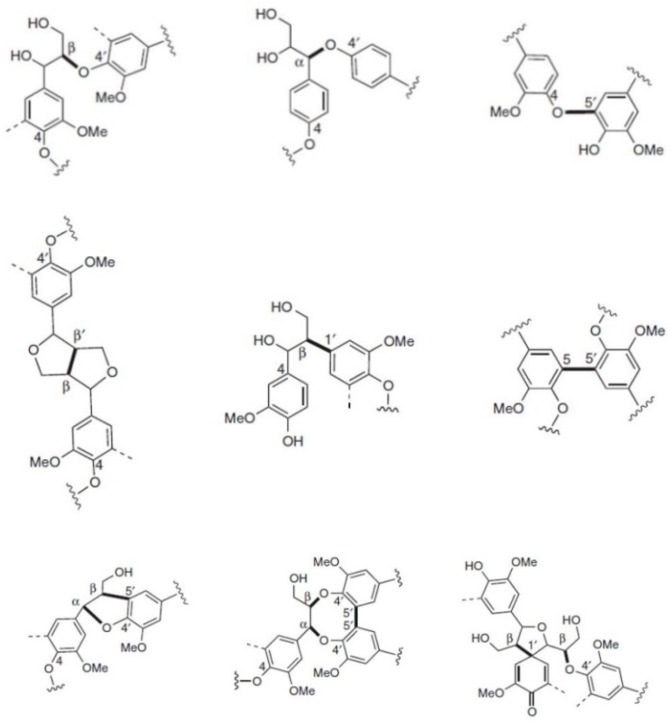
Most common monolignol linkages. First line: ether bonds (ß-O-4′, α-O-4′, 4-O-5′); second line: C–C bonds (ß-ß′, ß-1′, 5-5′), and third line: more complex linkages (ß-5′/ α-O-4′, 5-5′/ ß-O-4′/ α-O-4′, ß-1′/ ß-O-4′). Reprinted from [15] under open access license.

As a polyphenol, lignin has the potential as an antioxidant to prevent oxidation reactions in biofuels, animal feeds, and polymeric composite materials. Structurally, lignin is a randomly crosslinked polymer consisting of three different phenylpropane derivatives mainly linked by ether bonds (Figure 1 and Figure 2). Numerous studies were reported to elucidate the detailed 3D structure of lignin, including the formation of more complex bonds (as shown in Figure 2, last row) during biosynthesis, comprehensively reviewed by Lupoi et al. [18].

Due to this rather complex mixture of differently connected monolignols, most of the properties of lignins are dependent on the final 3D structure. Antioxidant capacity mainly is caused by hydrogen transfer and single electron transfer reactions and the capacity varies as soon as the number of available functional groups, such as OH and OCH_3_ changes. So far, lignin is not transferred to chemical industry as a commercial antioxidant due to this inhomogeneity [19,20]. Very recently, it was shown that plant portions such as stem- and leaf-derived lignins significantly differ in their monolignol composition and connectivity [21,22]. In addition to their UV absorption properties, the free radical scavenging ability of phenolic groups gives lignin an excellent antioxidant property and can increase thermal and oxidation stability of polymers in blends [23]. Sugarcane bagasse lignin and modified lignins can serve as natural, safer and less expensive potential antioxidant substitutes for synthetic antioxidants such as BHT. Also, unmodified lignin from sugarcane bagasse and epoxy lignin could be used as natural antibacterials [24].

For a long time, natural cellulose and related composites have been prepared and applied in many different fields including packaging, optics, and sensor technologies. Within the last five years, first examples of lignin/cellulose-based coatings have been reported [25]. In the context of food preservation, few studies have been reported on the use of organic active principles (vegetal) against bacteria/fungi plant pathogens, while a large amount of literature is available on how these active agents can be incorporated in biobased polymeric matrices. It is important to remember how crop damage and the economic losses of agriculture production caused by plant pathogens both represent a serious worldwide problem that needs innovative and green strategies able to preserve important fresh productions during their transport and storage. Recently, the possibility of obtaining various functionalities by using the combination of different nanofillers, including antimicrobial activity, was also considered; for example, cellulose nanocrystals and lignin nanoparticles were added to PLA films to both act as promising bioactive packaging for the preservation of fresh food products against food borne pathogens and to reinforce the PLA nanocomposites [26].

Among the many (polysaccharidic) biopolymers used for the preparation of edible films and coatings, hydroxypropylmethylcellulose (HPMC) has been the focus of many studies due to its availability, edibility, good film-forming properties (resulting in transparent films with suitable mechanical performance) and excellent gas and grease barrier properties, as well as its ability to retain a large amount of active compounds [27].

Chitosan, composed of 2-glucosamine and *N*-acetyl-2-glucosamine monomer units, is mainly produced from crustacean shells (i.e., crab, shrimp). Chitosans are biodegradable and biocompatible, furthermore they are attractive due to their antioxidant and antimicrobial activity. However, some disadvantages, such as dissolution in highly acidic solutions, low surface area, high cost, and poor thermal and mechanical properties, requires the combination with other components such as polyvinyl alcohol (PVA) to overcome the weaknesses [28].

In this work, lignin and chitosan were introduced in both binary (HPMC/lignin) and ternary (HPMC/lignin/chitosan) composite systems with different lignin types and weight loading using a solvent casting approach. The antiradical and antimicrobial activities of the prepared films were studied as well as the antimicrobial activities of added lignins.

## 2. Materials and Methods

### 2.1. Lignin Isolation and Purification

Industrial black liquor was delivered by from the Zellstoffwerk Blankenstein GmbH (Blankenstein, Germany). Gradual acidic precipitation of black liquor was performed for lignin extraction using HCl and H_2_SO_4_ with varying pH, temperature, and time of stirring. Corresponding yields were determined. L1 was extracted under the following conditions: H_2_SO_4_ with stirring at room temperature, pH = 2 for 90–180 min L2 was prepared via soaking of L1 with diethyl ether. Selective extraction with acetone and ethanol, respectively, produced L3 and L4. Diethyl ether was used to precipitate all samples. Monitoring was performed via thin layer chromatography (TLC). The organosolv lignins were isolated from spruce/pine (L5), beech wood (L6), and Miscanthus (L7) according to an earlier published procedure [29].

### 2.2. Lignin Purity, Ash and Sugar Content via NREL Measurements

The chemical composition (%, *w*/*w*) was determined according to the standard analytical procedures published by National Renewable Energy Laboratory (NREL) [30]. NREL measurements were performed by the BIOPOS Research Institute (Teltow-Seehof, Germany). HPLC analysis was conducted using water at a flow rate of 0.4 mL/min in a column (300 × 7.8 mm, Machery-Nagel, Düren, Germany) at a constant temperature of 90 °C. Structural carbohydrates in biomass and lignin samples were determined following NREL procedures [31,32,33,34].

### 2.3. 2D Heteronuclear Single Quantum Coherence (HSQC) NMR Analysis

According to the procedure described in [29], HSQC spectra were recorded with a spectral width of 7211 Hz, a receiver gain of 2050, and a total acquisition time of 0.28 s. O1 was set to 5 ppm (^1^H) and 80 ppm (^13^C).

### 2.4. ^31^P NMR Analysis

Analogue to a procedure given in [29], ^31^P NMR spectra were obtained using ^1^H-^31^P decoupling experiment (Avance III 600, Bruker, Karlsruhe, Germany), 131,000 points were recorded at 12,175.324 Hz.

### 2.5. Antioxidant Activity (DPPH Assay)

The DPPH inhibition was assessed by the procedure described by Alzagameem et al. using a Jasco V-630 spectrophotometer [13]. Shortly, dioxane/water (90:10, *v*/*v*) at a concentration of 1 g/L was used to dissolve the samples; a mixture was prepared of 0.1 mL of the sample and 3.9 mL of a 6 × 10^−5^ M DPPH solution. Finally, the absorbance was determined after 15 and 30 min, resp., at a wavelength of 518 nm.

### 2.6. Antimicrobial Activity of Lignins (Zone of Inhibition Test)

Inoculums of *Staphylococcus aureus* ssp. *Aureus* (DSM No 799), *L. monocytogenes* (DSM No 19094), and *E. coli* (DSM No 1576) were prepared by transferring a frozen culture to 10 mL of nutrient broth (Roth, Karlsruhe, Germany). Afterwards the broth was incubated at 37 °C for 24 h. In the beginning of each trial, the inoculum was diluted in physiological saline solution with tryptone (Oxoid, Hampshire, UK) to a final concentration of 10^7^ cfu/mL. 1 mL of the suspension was spread with a sterile spatula over the surface of a plate count agar plate (Roth, Karlsruhe, Germany). Three filter papers were impregnated with the antimicrobial agent, 0.1 g of lignin was dissolved in 1 mL DMSO and dried over a circular filter paper (0.5 cm of diameter) and three blank filter papers as references were applied on the inoculated agar plate. The agar plates were incubated for 24 h at 37 °C. A clear zone without microorganism growth (zone of inhibition) is related to the level of antimicrobial activity of the agent.

### 2.7. Film Formation

HPMC film-forming solutions were prepared using the procedure described by Sebti et al. by dissolving 3 parts of HPMC in 200 parts of 0.01 mol L^−1^ HCl solution, 100 parts of absolute ethanol, and 10% (w/w HPMC) of PEG 400 [35]. Twenty-five mL of the solution was plated onto a glass and dried at room temperature (RT) for 36 h. HPMC-lignin film-forming solutions were prepared by dissolving 5, 10, 15, 20, 25, and 30% (w/w HPMC) of the first fraction of the purification of kraft lignin (L1) in the smallest amount of DMSO then added to the HPMC-film forming solution mentioned before, and stirred for 15 min at RT. 25 mL of the polymer solution was plated onto a glass and dried at RT for 36 h. HPMC-Organosolv lignin (L5, L6 and L7) samples were prepared the same way. HPMC-lignin-chitosan film-forming solutions were prepared by dissolving 5% of 85% deacetylated chitosan in absolute ethanol and added to the HPMC film forming solution, while stirring, 5, 10, 15, 20, 25, and 30% of (L1, L5, L6, L7) dissolved in the smallest amount of DMSO was added to the HPMC-chitosan film forming solution, and stirred for 15 min at RT. Twenty-five mL of the polymer solution were plated onto a glass and dried at RT for 36 h.

### 2.8. Antioxidant Activity (DPPH Test)

The DPPH radical scavenging activity for the studied films was determined according to the method proposed by Yang et al., with a slight modification [12]. Films (0.1 g) were cut into small pieces and immersed in 2 mL of methanol for 24 h at room temperature then centrifuged for 2 min. The obtained supernatant was analyzed for evaluation of DPPH radical scavenging activity: an aliquot of methanol extract (0.5 mL) was mixed with 0.5 mL of DPPH in methanol (50 mg L^−1^). The mixture was maintained at room temperature in the dark for 60 min. The absorbance was measured at 517 nm using a UV–Vis spectrometer (Jasco V-630 spectrophotometer, Silver Spring, MD, USA). The mixture solution of methanol extracted from neat HPMC and DPPH methanol was used as control. DPPH radical scavenging activity was calculated by using Equation (1):

(RSA, %) = [(Acontrol − Asample)/ Acontrol] × 100(1)

where Asample was the absorbance of sample and Acontrol was the absorbance of the control.

### 2.9. Antimicrobial Activity of the Films

The antimicrobial activity of the samples was analyzed by modifying the Japanese Industrial Standard (JIS) Z 2801:2000 in order to test conditions typical for perishable products. The JIS is based on a comparison of bacteria counts (*S. aureus*, *E. coli*) in saline solution on reference and sample materials after a defined storage temperature and time (35 °C, 24 h) [36,37]. The material that shows a calculated log10-reduction ≥ 2.0 log10 units after 24 h is considered as an effective antimicrobial agent (JIS Z 2801:2000). If log10-reduction Ø ≥ 2 log10 units is reached, the antimicrobial activity against *B. thermosphacta* and *P. fluorescens* is then tested. The test will be conducted at a constant temperature of 7 °C for 24 h. In case the Ø log10-reduction is ≥ 2 log10, highly concentrated meat extract solution (18 μg mL^−1^) will be chosen as the reference media for perishable foods [36,37,38,39,40].

## 3. Results and Discussion

### 3.1. Antimicrobial Activity of Lignin

Lignin purification fractions were tested against both Gram-positive (*S. aureus* and *L. monocytogenes*) and Gram-negative bacteria (*E. coli*) to test the effect of purification on the antibacterial activity. Unmodified lignins were found to be the most effective against *Bacillus* sp. (Gram-positive bacteria) than against *Klebsiella* sp. (Gram-negative bacteria) [24]. Rocca and others discussed the antibacterial activity of different lignins and nanocomposites containing lignin that have higher sugar content against *S. aureus* (known to present a thicker peptidoglycan layer) as a representative of Gram-positive bacteria, and *E. coli* (contains more fatty acids) for Gram-negative. They concluded that the interaction of the nanocomposites with the bacterial cell wall can be governed by the lignin structure helping not only the stability of the particles but also their selectivity towards different types of bacteria [41]. The disk diffusion method was applied on lignin purification fractions L1 to L4. The samples were dissolved first in ethanol and tested, then in acetone and tested, and finally in DMSO and tested. The results showed that the inhibition zone of L1 increased from ethanol to acetone to DMSO. L2, L3 and L4 show no activity at all using ethanol and acetone while L2 showed slight activity against *L. monocytogenes* when dissolved in DMSO. This is due to the solubility of lignin in each solvent, where it is 100% soluble in DMSO, 60% soluble in acetone, and 40% soluble in ethanol. This allows a small fraction to dissolve, causing the antibacterial activity.

The effect of storage on the antibacterial activity of L1 was also studied, where a fresh sample of L1 was tested quantitatively against *S. aureus*. A fresh sample of L1 with a concentration of 0.1 g mL^−1^ gave a log10 reduction of 4.4, and 2.1 at the concentration of 0.001 g mL^−1^. After storing L1 for 6 months, L1 showed better activity with a log10 reduction of 4.5 at 0.1 g mL^−1^ and remained the same at 0.001 g mL^−1^. This is demonstrated in Alzagameem et al., 2018 through the analytical analysis of fresh and stored fractions and the DPPH inhibitions as well as the total phenol contents of the studied fractions [13]. Lignin degradation by time produces low molecular weight species that have both antioxidant and antibacterial capacities.

Based on the effect of solvent on the antibacterial activity, further studies on lignin samples were done using DMSO to guarantee that all active species were dissolved and introduced to the tested platelets. Table 1 shows the result of the disk diffusion method of the studied purification fractions in addition to two softwood-based organosolv lignins: from spruce/pine (L5) and from beech (L6) as well as a grass-based organosolv lignin: from Miscanthus L7 for comparison purposes.

The reference, which does not contain lignin, does not have any observed inhibiting effects on the bacteria, but is overgrown with colonies. *L. monocytogenes* has a higher antimicrobial efficacy than *S. aureus*. This can be seen by L1, L2, L5, and L6, in which inhibition zones around the platelets have formed on the *L. monocytogenes* plates. The tests show, as well, that the first fraction of the purification (L1 to L4) has the highest activity. Organosolv lignins obtained from softwood have certain activities while the lignin obtained from grass L7 has none. The beech based (L6) sample specifically has higher activity that the spruce/pine-based samples (L5); it actually has the highest activity among all the lignins studied. L1, on the other hand, as a kraft lignin, has higher activity than L5. The same with Organosolv lignins: Gram-positive bacteria were inhibited more than Gram-negative. In regard to *E. coli*, all studied lignins show no activity at all.

The antimicrobial mechanism may involve the generation of localized heat and reactive oxygen species (ROS) upon light irradiation. Rocca et al. postulated the sugar content of the lignin might cause and/or support the adhesion to the bacterial membrane. For example, the peptidoglycan layer of bacterial cell walls is able interact with sugar molecules, thereby increasing the activity against *S. aureus* [41]. The NREL results of the lignins L1 to L4 in Table 2 show that L1 has the lowest acid insoluble lignin content and the highest acid soluble content. Glucan content (polysaccharide) is the highest for L1. Also, only L1 and L2 have arabinan content. Xylan content for L1 and L2 as well was much higher than that for L3 and L4, but it seems that the effect of the acid soluble lignin content has the main role in the antibacterial activity since L1 was more active than L2 and it correlates with the antibacterial activity results. This supports the finding of Rocca et al. [41]. PH2 SK was extracted according to Klein et al., 2018 [15] at pH = 2 which has almost the same NREL values as L2.

**Figure 3:**
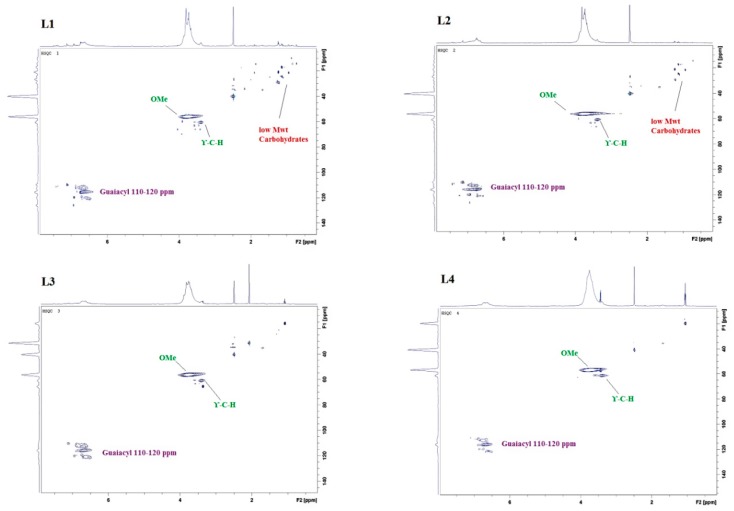
HSQC of lignin purification fractions: L1, L2, L3, and L4.

The HSQC analysis of the purification fractions in Figure 3 shows low molecular weight species in L1 at chemical shift δC/δH 10.0–37.0/2.5–0.7. The number of those species decreased going through the purification from L1 to L4. It indicates the carbohydrate content which is also supported by the NREL testing.

In addition, it was recently shown that 2D HSQC NMR in combination with SEC measurements can be used to determine the molecular weights (Mw) of biopolymers using multivariate data analysis (i.e., principal component analysis) to simulate Mw data [42]. The ^31^P NMR analysis, on the other hand, shows the presence of aliphatic OH groups in all the purification fractions confirming data reported for lignins obtained from other sources and isolated using different methods [43,44,45].

The number of OH groups in the lignin fractions (Table 3) was obtained from the ^31^P NMR analysis, showing that the aliphatic OH number of the L1 samples was the highest, followed by L2, L3, and L4 which have half this number. Also, the number of the carboxylic acid OH groups for L1 was also the highest, demonstrating the antibacterial activity as it is usually related to its origin, and specifically due to the presence of phenolic compounds and different functional groups containing oxygen (methoxyl and epoxy groups) in its structure [18,19,24]. PH2SK was extracted according to Klein et al., 2018 [15]. Previous studies confirmed the degradation of lignin with temperature around 60 °C [13,19]. During the extraction of PH2SK, the black liquor was heated to 50–60 °C. The heating could have produced phenolic carboxylic acids, containing fragments which are demonstrated by the high number of phenolic carboxylic acid OH groups in Table 3. Primary antimicrobial activity tests showed certain activity of PH2SK against Gram-positive bacteria which also matched with the NREL results.

In a previous study, X-ray diffraction (XRD) measurements confirmed the increase of the purification level from L1 to L4 [13]. For kraft lignin samples purified by selective extraction, the storage effects were observed to result in depolymerization being initiated by temperature and UV irradiation, respectively [46,47].

### 3.2. Preparation of Lignin-Derived Composites

Film preparation was based on the antibacterial activity results as well as the antioxidant activities of the lignins; the polymerization of HPMC-based films was limited to the selected active lignin samples: L1, L5, and L6 samples to be tested against Gram-positive and Gram-negative bacteria. The antioxidant activity of the lignins was studied using the DPPH assay [11]. Table 4 shows the DPPH inhibitions of the lignin samples. All of the lignin samples show a specific activity that varies from high (68.2% of L4) to low (31% of L7). Accordingly, lignins from L1 to L7 were added to HPMC and the antioxidant activity of the resulted films was then investigated.

**Figure 4:**
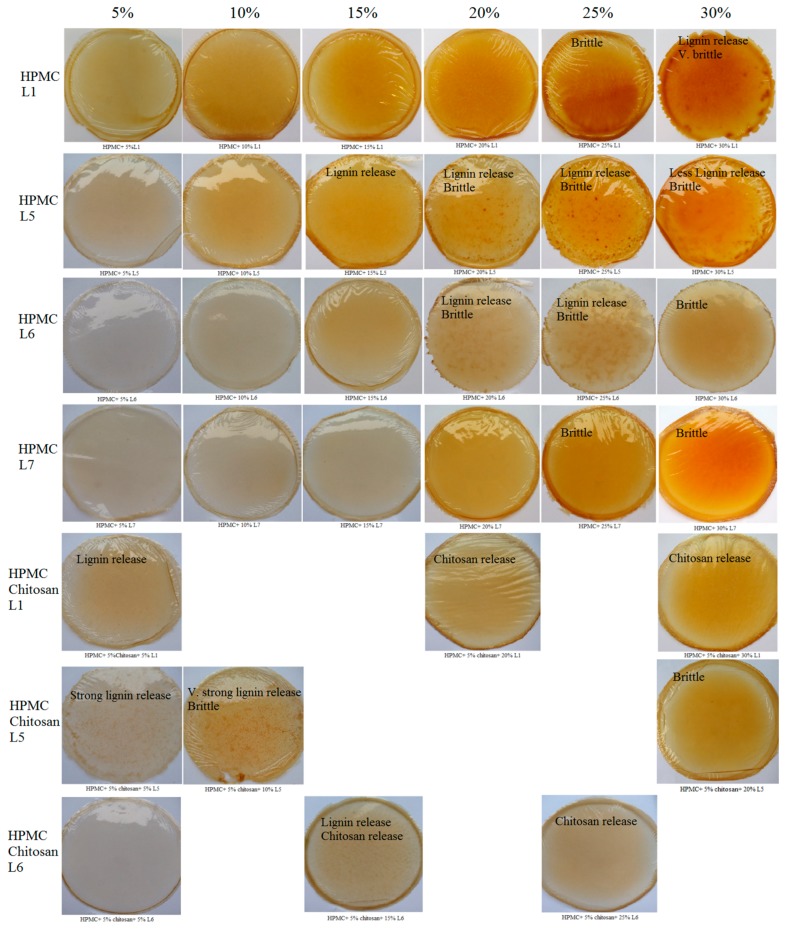
HPMC/lignin and HPMC/lignin/chitosan composite films.

The lignin was added to the HPMC during polymerization in addition to polyethylene glycol 400 (PEG) that improved the flexibility and impact strength of the blend and act as a compatibilizer with the polymer. As mentioned, the polymers were produced based on the antioxidant activities as well as the antibacterial activities of the lignins (L1 to L7). HPMC-film is transparent and water soluble. Water solubility decreases with the addition of lignin. The color of the films was light honey and became denser with the addition of lignin as shown in Figure 4. The brittleness appeared after the addition of 25% of the total weight of the sample in lignin to both L1 and L7, at 30% of L5, and at 20% of L6. Lignin release started at 30% of L1 and 15% of L5, while L7 films did not exhibit any release. L6 had lignin release at 20% and 25% but had none at 30%. Primary antimicrobial tests on the films against Gram-positive bacteria confirmed the activity of the films against *S. aureus*. The films were not active against *E. coli*, and hence, the addition of chitosan was the key. Eighty-five percent chitosan (deacetylated) was added to the HPMC-lignin film solutions in 5% increments, the lignin additions to the HPMC and chitosan were chosen based on the best lignin-HPMC combination with no lignin release and no brittleness. Lignin release appeared at lower percentages starting at 5% in L1 films, at 5% and 10% in L5 films, and at 15% in L6 films, while chitosan release happened at 20% and 30% for L1 and at 15% and 25% for L6 while HPMC-L5-chitosan films exhibited no chitosan release. The lignin and/or chitosan release depended on the compatibility of the three components in the film in addition to the lignin type. In HPMC-lignin films, the increment of the lignin added to the HPMC solution improved the mechanical properties of the HPMC film and decreased the water solubility. The lignin release for the softwood based organosolv lignins started at lower lignin percentages compared to kraft lignin and grass-based organosolv lignin. The HPMC-lignin-chitosan films are more complex: the lignin started the release at lower concentrations while the chitosan started the release at higher lignin concentration. The released lignin/chitosan were non-reacted species that failed in the competition to bond in the film cluster. At low lignin concentrations, the chitosan won the competition and bonded fully in the film cluster, leaving the rest of the lignin to release. At high lignin concentrations, the lignin won the competition and bonded fully to the film cluster, leaving the rest of the chitosan out of the polymer structure. This may be due to the increased number of the phenolic OH groups introduced by the lignin at higher concentrations.

### 3.3. Antiradical Activity of Lignin-Derived Films

**Figure 5:**
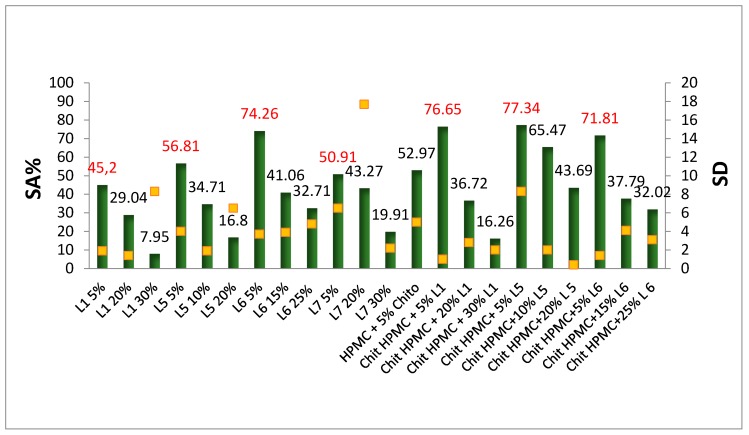
Antioxidant activity of HMPC/lignin and HPMC/lignin/chitosan films. The measurements were held in triplets. The red data labels indicate the highest activity for each lignin type. The yellow points relate to the standard deviation.

Radicals originating from oxygen exist naturally in the atmosphere or can be created by the thermal processing or irradiation of packaging and food. These radicals act as initiators of the chain oxidation of lipids. It is therefore advantageous to eliminate theses radicals from the headspace and the bulk of the food, as an alternative route to eliminate oxygen from the package. The radical scavenging efficiency of an antiradical substance depends on the rate of hydrogen atom abstraction from the phenyl group and also on the stability of the resulting radical. Strategies employed in active packaging generally include: (1) the design of active compound releasing systems and (2) undesired compound scavenging systems. In the first strategy, the generation of low molecular weight substances inside the packaging film and promotion of their migration into the food stuff is of interest. In the second strategy, one of the advantages of radical scavengers is their efficiency upon contact without the need for the release of active compounds. This has been shown for hydroxyl radicals in the gas phase scavenged by essential oils supported on active packaging films containing essential oils [12]. The phenolic mobile hydrogen atom (ArO–H) plays the main role in the ability of monomeric phenolic compounds to scavenge free radicals (R •). The process occurs according to the following scheme: ArO–H + R • → ArO • + R–H [13]. In the plant cell wall, lignin is often associated with carbohydrate polymers. The nonphenolic carbohydrate impurities, which remain strongly associated with lignin during its isolation and purification, can decrease the concentration of active phenolic OH groups and negatively influence their reactivity (by increasing the O–H bond dissociation enthalpy). The phenoxy radicals in some lignins can take part in the reaction with oxidant radicals as secondary antioxidants [45]. Figure 5 shows the scavenging activity (SA) of free radical DPPH of the HPMC-based films. The films studied depended on the percentage of the lignin added; the lowest addition (5%), the highest addition (30%), and the lignin addition that produces the best film for each lignin sample.

The results showed that the 5% addition of lignin had the highest SA% and the 30% had the lowest in which the UV absorption increased from the 5% to the 30% and the SA decreased. During the polymerization, lignin reacted with HPMC to form ether or ester bonds when 5% of lignin was added. Lignin is reactive to an extent where some phenolic OH groups are left free, causing the SA; however, not every phenolic OH group takes part in the reaction with the free radical [48]. For the 30% addition, the high concentration of lignin increased the connectivity of HPMC-lignin, where it converted the phenolic OH groups to an ether or ester bond or even self-reacted; this decreased the number of active phenolic OH groups against oxidants and hence decreased the SA. Chitosan-lignin-HPMC films, in general, have higher SA than lignin-HPMC films because simply, the activity of neat chitosan, which is basically due to the hydroxyl and the amine groups [49], was added to the lignin-HPMC activity; also, the reaction between the components could happen on the α-position of the phenolic group in lignin, where an electron donating group (like methyl or methylene) increases the SA and an electron withdrawing group (like carbonyl or carboxyl) decreases the SA by increasing the polarization of the –OH bond and complicating its hemolytic dissociation [50,51]. The type of lignin added also affects the SA value. The trend of the SA of all the samples, based on the lignin type, was: organosolv of softwood > kraft of softwood > Organosolv of grass. The Organosolv lignins, in general, had a higher methoxyl content than kraft lignins which may act as a secondary antiradical and the carbohydrates content is higher in kraft lignin than organosolv [52], resulting in a decreased SA value. Table 4 shows the DPPH inhibition of the added lignins, where L1 has the highest and grass-based organosolv lignin has lowest. This basically could be the reason why films with lignins from grass have lower antioxidant activity.

Small angle X-ray scattering (SAXS) studies are ongoing to obtain more detailed information regarding the film morphology including micro- and/or nanostructures as observed for lignin-derived polyurethanes [16,53].

### 3.4. Antimicrobial Activity of HPMC/Lignin and HPMC/Lignin/Chitosan Composites

**Figure 6:**
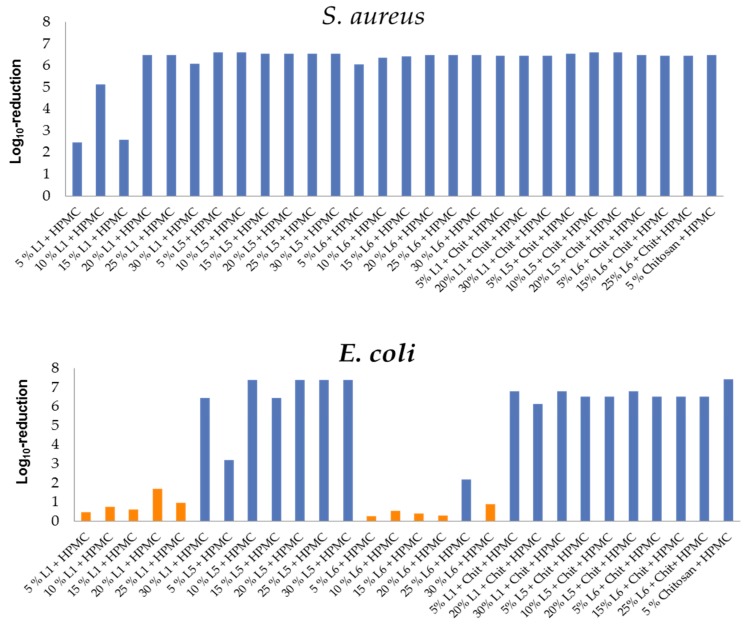
Antimicrobial activity of HMPC/lignin and HPMC/lignin/chitosan films against *S. aureus* (above) and *E. coli* (below). All films are active against *S. aureus*. Columns in orange relate to nonactive films against *E. coli*. Chitosan incorporation increased the activity. L5 films showed highest activities against both bacteria.

**Figure 7:**
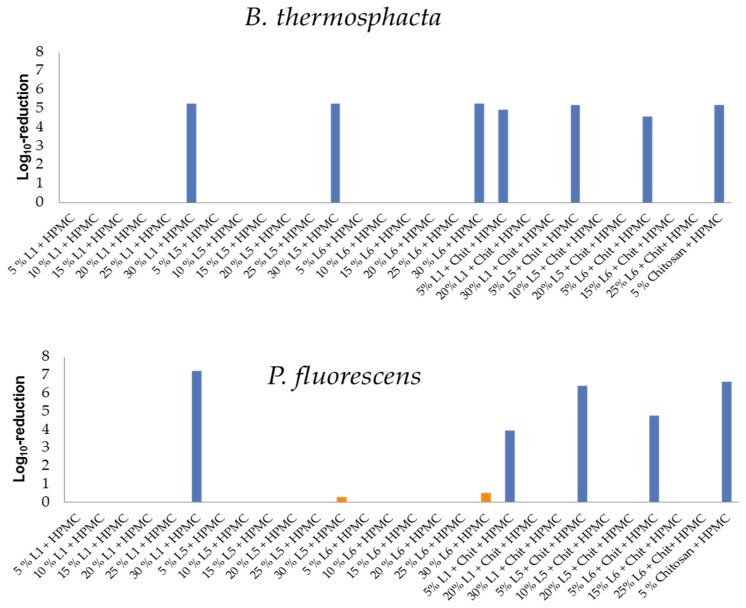
Antimicrobial activity of HMPC/lignin and HPMC/lignin/chitosan films against *B. thermosphacta* (above) and *P. fluorescens* (below). Columns in orange relate to nonactive films against *P. fluorescens*.

The prepared films were tested against *S. aureus* and *E. coli* at 35 °C for 24 h in pure culture. In regard to Dohlen and coworkers, those films that reached a log10-reduction Ø ≥ 2 log10 were tested against *B. thermosphacta* (a Gram-positive bacteria that is one of the most abundant spoilage organisms of fresh and cured meats, fish, and fish products) due to its tolerance to high-salt and low-pH conditions, its ability to grow at refrigeration temperatures (4 °C), and its production of organoleptically unpleasant compounds and *P. fluorescens* (Gram-negative bacteria that poses a significant spoilage problem in refrigerated (0–7 °C) meat and dairy products) [38,54]. A log10-reduction Ø ≥ 2 log10 units allowed the adaption of temperature and inoculated solution. The test was conducted at a constant temperature of 7 °C for 24 h. In case the Ø log10-reduction was ≥ 2 log10, highly concentrated meat extract solution (18 μg mL^−1^) will be chosen as the reference media for perishable foods. Figure 6 shows the antimicrobial activities of the studied films against *S. aureus* and *E. coli*; Figure 7 shows them against spoilage bacteria at low temperatures (*B. thermosphacta* and *P. fluorescens*).

The results in Figure 6 correlate with the inhibition zone test results (Table 1), in which lignin films are active against Gram-positive bacteria more than Gram-negative bacteria. For *S. aureus*, the activity of films containing organosolv lignins in general was higher than kraft lignins. This could be because the carbohydrates in the kraft lignin could have reacted with the HPMC and entered the film structure, leaving the film without the active part of the sugar content that participated in the antimicrobial activity of the lignin alone. The activity of the lignin films increased with increasing lignin concentration in the film which introduced more active functional groups (aliphatic OH, carbonyl CO, COOH) into the film structure. For *E. coli*, the kraft lignin films exhibited no activity at low concentration. Only the 30% addition had an activity, which is justified by the introduction of more active functional groups into the film and the lignin release could be the main reason of the activity. HPMC-L5 films exhibited high activity at higher percentages, which also could be enhanced by the released lignins; the 5% addition had the lowest activity due to the concentration factor. The 25% L6-HPMC film was the only active one among HPMC-L6 films. The chitosan addition to the HPMC-lignin combination improved the activity since chitosan is known as an antibacterial compound against both Gram-positive and Gram-negative bacteria. It gains its activity due to the presence of the amino group and the OH functionality in its structure [55,56].

The chitosan release did not have a significant effect on the samples and the activities of the chitosan films were close to each other. Also, the chitosan activity of the corresponding films dominates over the lignin activity. Thus, can be clearly seen from the chitosan-HPMC activity compared to the chitosan-HPMC-lignin activities, with the exception of the slight negative effect of lignin on the activity of the films in which more amino or OH groups of chitosan and/or lignin are involved in bonding with the HPMC blocking the antimicrobial activity for some of those functionalities.

The films were further tested against spoilage bacteria that grow at low temperatures (0–7 °C): *B. thermosphacta* (Gram-positive) and *P. fluorescens* (Gram-negative). Figure 7 shows that the 30% addition of all lignins in HPMC-lignin films was active against *B. thermosphacta*. On the other hand, the 5% addition of the lignins in the HPMC-lignin-chitosan films was the active one. The activities were close to each other and showed no effect of the lignin type.

In HPMC-lignin films, the 30% addition of L1 is the only active film against *P. fluorescens*; L5 and L6 showed no activity. In HPMC-lignin-chitosan films, the 5% addition of L5 had the highest activity. Strangely, activity of L1 against *P. fluorescens* was higher than that against *B. thermosphacta*; this may be due to the lignin release (which has to be clarified in ongoing studies).

Differences in the level of antimicrobial activity could be explained by a variety of factors, such as the Gram characteristics of the bacteria, the ability of building exopolysaccharides, differences in the zeta potential, differences in the permeability of the outer membrane which itself is influenced by temperature, different fatty acids, etc. These factors combined with different charges of the surfaces could have caused the variation in the antimicrobial activity against different bacteria. As an example, in different studies, a higher activity of high molecular weight chitosan against Gram-positive bacteria (*S. aureus*) compared with Gram-negative bacteria, *E. coli* and *Ps. fluorescens* was observed [36,37,39]. In our results, *P. fluorescens* is even more resistant than *E. coli*, which is due to higher production of exopolysaccharides by *P. fluorescens*.

## 4. Conclusions

In addition to antioxidant activity, lignins of different origins show distinct antimicrobial effects. Due to the obtained results, purification of kraft lignin via solvent extraction has a negative effect on the antimicrobial activity against *S. aureus* and *L. monocytogenes* most obviously due to the presence of carbohydrate content and aliphatic OH groups which was supported by both NREL and NMR analysis. The biomass source (hard and soft wood, grasses) and pulping process influence both antioxidant and antimicrobial activity. Organosolv lignins, in general, have a higher methoxyl content than kraft lignins which may act as a secondary antiradical and the carbohydrate content is higher in kraft lignin than organosolv. In addition, the antimicrobial activity of the lignins also depends on the solvent polarity (as shown for ethanol, acetone, and DMSO). Storage led to an increase in antimicrobial activity against *S. aureus* due to the degradation of lignin over time. The scavenging activity of both binary (HPMC/lignin) and ternary (HPMC/lignin/chitosan) systems was affected by the percentage of the added lignin. The lignin release in the produced films positively affected the activity and the chitosan addition enhances the activity even more for both Gram-positive and Gram-negative bacteria. Spoilage bacteria that grow at low temperatures, such as *B. thermosphacta* and *P. fluorescens*, were affected by lignin/chitosan composite films. The detailed mechanism regarding the bioactivity is the focus of ongoing studies and might be correlated to bacterial surfaces charges and zeta potential.

## Figures and Tables

**Table 1:** Inhibition zones of lignin samples against *S. aureus, L. monocytogenes*, and *E. coli* obtained by disk diffusion method.

Lignin Platelets	Bacteria	
	*S. aureus*	*L. monocytogenes*
**Reference** **DMSO**	− −1x contaminated with yeast	−−
**L1**	+ (max 1–2 mm)	++ (max 5 mm)
**L2**	−	+ (max 1 mm)
**L3**	−	+ (max 1 mm), slight growth in inhibition zone
**L4**	−	−
**L5**	+ (max 1 mm)	+ (max 1–2 mm)
**L6**	++ (max 2–3 mm)	+ (max 7 mm)
**L7**	−	−

−− = no inhibition, overgrowth of platelets; − = no inhibition; + = Inhibition; ++ = strong inhibition; max = maximum: measured at the greatest distance from the plate.

**Table 2:** Compositional analysis according to National Renewable Energy Laboratory (NREL) procedure of kraft lignin fractions (L1 to L4, purified via solvent extraction and PH2SK, isolated at pH2 without further purification).

Fraction	AIL [%]	ASL [%]	Total Lignin [%]	Ash [%]	Glucan [%]	Xylan [%]	Galactan [%]	Arabinan [%]	Rhamnan [%]	Mannan [%]	Sum [%]
**L1**	86.86	15.14	102.00	1.01	0.45	0.59	0.00	0.04	0.00	0.00	104.09
**L2**	93.37	9.76	103.13	0.88	0.31	0.76	0.00	0.06	0.00	0.00	105.15
**L3**	91.43	9.46	100.89	0.24	0.26	0.04	0.00	0.00	0.00	0.00	101.42
**L4**	95.17	2.42	97.59	0.40	0.36	0.07	0.00	0.00	0.00	0.00	98.42
**PH2SK**	86.24	13.28	99.52	0.85	0.26	0.85	0.00	0.07	0.00	0.00	101.55

AIL = acid insoluble lignin; ASL = acid soluble lignin.

**Table 3:** Number of OH in the lignin fractions as determined by ^31^P NMR analysis.

	Aliphatic OH	Condensed-OH	G and Dimethylated-OH	Carboxylic Acids-OH
**L1**	8.70	5.04	6.30	0.59
**L2**	8.52	0.28	0.71	0.00
**L3**	3.81	2.99	2.91	0.16
**L4**	3.17	0.48	1.79	0.15
L5 *	3.02	0.21	0.18	0.02
**PH2SK**	7.32	7.11	9.73	3.10

* L5 has S–OH: 0.01.

**Table 4:** The DPPH inhibitions of kraft lignin fractions (L1 to L4) and organosolv lignins obtained from spruce/pine (L5), beech (L6), and *Miscanthus x giganteus* (L7).

	L1	L2	L3	L4	L5	L6	L7
**DPPH Inhibition (%)**	65.1 ± 3.7	66.8 ± 6.6	62.2 ± 9.5	68.2 ± 3.6	42 ± 1.9	64 ± 2.6	31 ± 1.0
